# First report of free-ranging maned wolf (*Chrysocyon brachyurus*) coinfected with *Dioctophyma renale* and *Pearsonema* sp. in Brazil

**DOI:** 10.1007/s11259-026-11357-3

**Published:** 2026-06-19

**Authors:** Alexandre Carvalho Costa, Elisabeth Neves Mureb, Naiara Vidal Stocco, Ágatha Ferreira Xavier de Oliveira, Bruna Emely Pereira Barbosa, Cristiano Chaves Pessoa da Veiga, Andresa Guimarães, Juliana Macedo Raimundo, Huarrisson Azevedo Santos, Daniel de Almeida Balthazar, Cristiane Divan Baldani

**Affiliations:** 1 Instituto de Veterinária, Departamento de Medicina e Cirurgia Veterinária, Universidade Federal Rural do Rio de Janeiro – UFRRJ, Seropédica, Rio de Janeiro Brasil; 2 Zoonoses & One Health, Facultat de Veterinaria, Universitat Autònoma de Barcelona, Cerdanyola del Vallès, Barcelona Spain; 3 Instituto de Veterinária, Departamento de Microbiologia e Imunologia Veterinária, Universidade Federal Rural do Rio de Janeiro – UFRRJ, Seropédica, Rio de Janeiro Brasil; 4 Instituto de Veterinária, Departamento de Epidemiologia e Saúde Pública Veterinária, Universidade Federal Rural do Rio de Janeiro – UFRRJ, Seropédica, Rio de Janeiro Brasil

**Keywords:** Sedimentoscopy, Urinalysis, Parasitology, Morphometry, Morphology, *Pearsonema*

## Abstract

The manned wolf (*Chrysocyon brachyurus*) has a highly diversified diet, that includes fish and amphibians, which may harbor infective forms of parasites, such as *Dioctophyma renale* and *Pearsonema* (syn. *Capillaria*) spp. Both parasites affect the urinary tract of their hosts, with *D. renale* being capable of infecting a wide range of mammalian species, whereas *Pearsonema* spp. exclusively parasitizes wild and domestic canids and felids, releasing their eggs in the urine of the host. This study reports the first coinfection by *D. renale* and *Pearsonema* sp. in the urine of a free-ranging maned wolf as well as associated laboratory findings. A male adult maned wolf, injured in a train accident in an urban area, was referred to the Centro de Triagem de Animais Silvestres (CETAS) – Rio de Janeiro, Brazil, where hemogram, biochemistry, ultrasonography, and urinalysis were performed. Ultrasonography examination revealed findings consistent with parasitism by *D. renale* in the right kidney and right perirenal region. Urinary sediment examination showed *D. renale* (5/field) and *Pearsonema* sp. eggs (1/field). Egg morphometry was performed, yielding mean length and width values of 62.2 μm and 41.2 μm for *D. renale* and 58.0 μm and 26.1 μm for *Pearsonema* sp., respectively. Through sediment examination and morphometry, it was possible to confirm *Pearsonema* sp. and *D. renale* infection, the latter also confirmed by ultrasonography, highlighting the importance of urinalysis in wild animals and demonstrating the need for continuous investigation into *Pearsonema* spp. which are poorly studied in wild canids.

## Background

Maned wolves (*Chrysocyon brachyurus*) are neotropical mammals that inhabit western Peru, southern regions of the Amazon Forest in Bolivia, through northern Argentina and Paraguay, and into eastern Brazil and northern Uruguay (Paula and Dematteo 2015). The species’ ecology is characterized by behavioral traits that may favor disease transmission, such as relatively large home ranges, scent marking and omnivorous feeding habits, that include fish and amphibians (Motta-Junior et al. 1996). The maned wolf is a threatened species at multiple levels, is currently classified as ‘Near Threatened’ (NT) in the International Union for Conservation of Nature (IUCN) Red List (Paula and Dematteo 2015). Several factors, such as habitat loss and road mortality, threaten the viability of maned wolf populations, and considering that wild canids are susceptible to numerous infectious agents (Deem and Emmons 2005), serious threats for wild canid conservation should be considered.

Maned wolves are susceptible to several infectious and parasitic diseases (Deem and Emmons 2005). However, little is known about the long-term effects of urinary tract parasites in this species, since few studies have addressed this issue and affected these animals often die prematurely from other causes, particularly road accidents (Oliveira et al. 2021).

*Dioctophyma renale* GOEZE 1782 is a cosmopolitan nematode that parasitizes the urinary tract of wild and domestic mammals. Infection occurs through the ingestion of infected intermediate hosts (aquatic oligochaete annelids *Lumbriculus variegatus*) or paratenic hosts (amphibians and freshwater fish) (Pedrassani et al. 2009). In the definitive hosts, *D. renale* larva penetrates the duodenal wall, enters the abdominal cavity, and migrates to the kidney, where they remain until reaching adulthood and subsequently release eggs into the urine (Pedrassani et al. 2009). In Brazil, *D. renale* has been reported in several wild mammal species, including the maned wolf (*Chrysocyon brachyurus*) (Oliveira et al. 2021).

Capillariid nematodes of the genus *Pearsonema* (syn. *Capillaria*) infect the urinary tract of domestic and wild carnivorous and omnivorous mammals (Petersen et al. 2018; Tamaru et al. 2025). Infections caused by *P. plica*, *P. feliscati*, *P. mucronata*, *P. neoplica* n. sp., *P. iharai* n. sp. and *P. toriii* n. sp. have been documented in the urinary bladder, ureters, and renal pelvis of wild and domestic carnivores (Beldomenico et al. 2002; Tamaru et al. 2025). Infection occurs through ingestion of intermediate hosts (earthworms of the family Lumbricidae) infected with nematode larvae, which migrate from the small intestine to the urinary bladder, releasing their eggs in urine upon reaching sexual maturity (Beldomenico et al. 2002; Guimarães et al. 2020). Some authors have suggested that birds and rodents may serve as paratenic hosts involved in the transmission of these parasites (Petersen et al., 2018).

Studies on urinary tract parasitism in Neotropical carnivores are scarce. In the present study, a case of coinfection with *Dioctophyma renale* and urinary capillariosis, together with associated laboratory findings, in a free-ranging maned wolf (*Chrysocyon brachyurus*), is reported.

## Case presentation

**Fig. 1:**
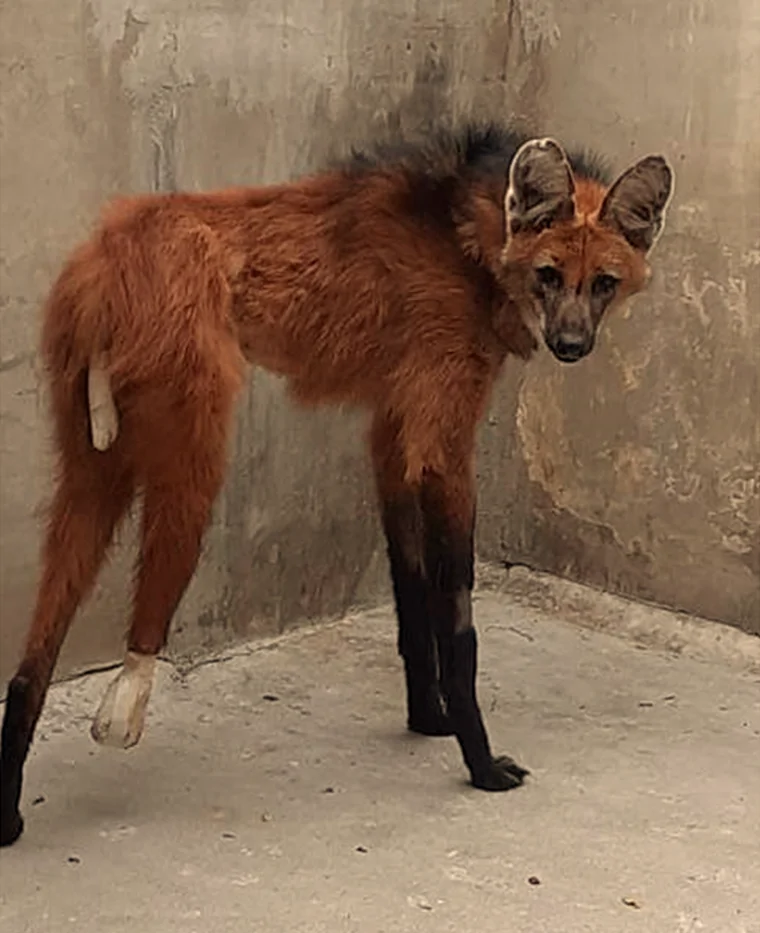
Maned wolf (*C. brachyurus*) following physical examination, presenting a traumatic amputation and an open fracture in the region of the right tarsometatarsal joint and middle third of the tail

In September 2020, an adult male maned wolf was rescued after being involved in a train collision in an urban area, and was subsequently referred to the Centro de Triagem de Animais Silvestres (CETAS-RJ) in Seropédica, Rio de Janeiro (22°72’35” S; 43°70’98” W). During the physical exam, apart from the traumatic amputation and open fracture in the right tarsometatarsal joint and middle third of the tail (Fig. 1), no other clinically significant abnormalities were observed. After stabilization, a blood sample (Day 1) was collected for hematological evaluation, and anemia, leukocytosis, neutrophilia, and monocytosis were observed (Table 1).

**Table 1:** Hematological results obtained from a maned wolf (*C. brachyurus*) presenting traumatic amputation and exposed fracture in the region of the right tarsometatarsal joint and middle third of the tail

Parameters	Day 1	Day 5	Reference (May-Junior et al. 2009)
RBC (x10^6^/µL)	3.77	2.24	4.10–5.90
Hemoglobin (g/dL)	10.4	6.0	10.7–15.4
PCV (%)	31.8	18.5	34.0–48.0
MCV (fL)	84.4	82.6	76.0–89.0
MCHC (g/dL)	32.7	32.4	30.0–34.0
WBC (/µL)	25,300	14,100	7900–19,100
Neutrophilic Bands (/µL)	253	141	0–510
Neutrophils (/µL)	22,011	8883	5500–16,100
Lymphocytes (/µL)	1012	2115	800–3700
Monocytes (/µL)	1518	1833	100–1300
Eosinophils (/µL)	253	1128	0–1700
Basophils (/µL)	253	0	Rare
Platelets (/µL)	240,000	324,000	150,000–500,000
TPP (g/dL)	5.8	6.2	5.5–8.0

*RBC* Red Blood Cells, *PCV *Packet Cell Volume, *MCV* Mean Corpuscular Volume, *MCHC *Mean Corpuscular Hemoglobin Concentration, *WBC *White Blood Cells, *TPP *Total Plasma Protein

Abdominal ultrasonography revealed an elongated tubular echogenic structure with a double hyperechoic wall within the right kidney, measuring approximately 1 cm in diameter, and compatible with the parasite *Dioctophyma renale*. The parasite was located within the pyelocaliceal system and extended into the right perirenal region. The right renal parenchyma was thinned, with loss of corticomedullary differentiation and reduced echogenicity, findings consistent with chronic parasitic nephritis and partial tissue destruction. The contralateral kidney showed no remarkable abnormalities.

The urinary bladder was moderately distended, contained a small amount of fine echogenic sediment suspended within the lumen and deposited on the bladder floor, with no evidence of mineralized calculi. The bladder wall appeared normal. No ultrasonographic abnormalities were observed in the remaining abdominal organs. The ultrasonographic findings were consistent with *Dioctophyma renale* parasitism in the right kidney and right perirenal region, as well as mild urinary bladder sedimentation. No diagnostic images were obtained during the examination.

Subsequently, a new blood count (Day 5), as well as serum biochemical analyses and urinalysis obtained through cystocentesis, were conducted. Hematological evaluation revealed anemia and monocytosis (Table 1), and the results of serum biochemistry (albumin, alanine aminotransferase, creatinine, alkaline phosphatase, glucose, globulins, total protein, and urea), remained within the normal limits considered for the species (May-Junior et al. 2009).

**Fig. 2:**
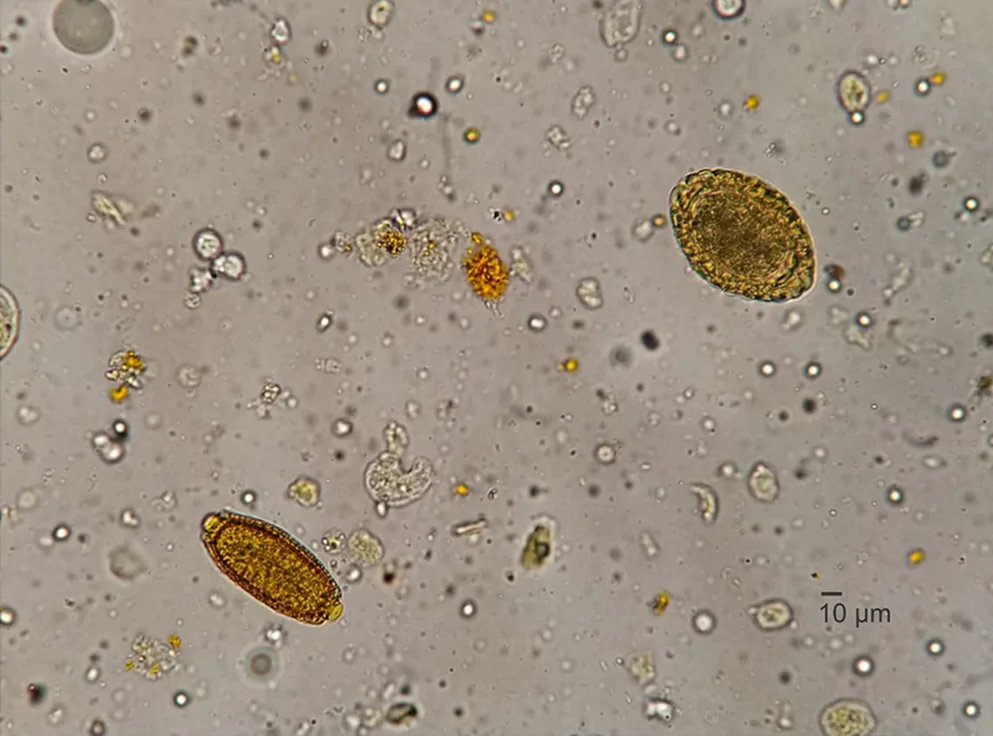
Eggs of *Pearsonema* sp. (left) and *Dioctophyma renale* (right) found in the urinary sediment of the maned wolf (*C. brachyurus*)

The urine was golden yellow and turbid, with a specific gravity of 1.037. Chemical analysis showed a pH of 6.0, a strong presence of blood (3+), and a slight presence of protein (1+), while all other parameters were negative. Urinary sediment examination revealed five *Dioctophyma renale* and one capillariid egg per field (Fig. 2), measuring 60.4-65.7 × 38.9–42.6 μm (mean 62.2 × 41.2 μm / *n* = 10) and 49-67 × 22.4–29.8 μm (mean 58 × 26.1 μm / *n* = 10), respectively. As shown in Fig. 2, *D.* *renale* eggs had an elliptical shape, symmetrical bipolar plugs and a thick, rough shell, while *Pearsonema* eggs presented a barrel shape, thick, and lightly pitted shell, bioperculate, and dark brown color as well as described in literature (Measures 2001; Guimarães et al. 2020). Other findings included hematuria (51 erythrocytes/field), leukocyturia (11 leukocytes/field), bilirubin crystals (2+), desquamated epithelial cells (1+), from the pelvis (2+) and transitional (3+), bacteria (cocci) (2+), and bilirubin staining (2+). The urinary protein/creatinine ratio was 0.12.

Following clinical stabilization, nephrectomy and orthopedic fracture repair were planned. However, before these procedures could be performed, the animal was transferred to another institution as part of an agreement between the institutions involved and in full compliance with the regulations and requirements established by relevant environmental authorities. Therefore, unfortunately, it was not possible to monitor its clinical progression or obtain information regarding the outcome of the case after transfer.

### Discussion and conclusions

The maned wolf in the present study was injured in a train collision in an urban area. As reported in previous studies, free-ranging animals are vulnerable traumatic injuries resulting from habitat degradation, proximity to urban centers, conflicts with rural producers (Hammond 2012), and especially, vehicle collisions, which represent one of the leading causes of significant population loss for this species (Oliveira et al. 2021).

The anemia and neutrophilic leukocytosis observed were likely associated with hemorrhage and inflammation caused by the traumatic injuries. Monocytosis was consistent with tissue repair and inflammatory response (Grzelak and Fry 2022).

The irreparable right renal lesion observed in this study was due to complete parenchymal destruction caused by *D. renale*, and because of the loss of renal function, this condition may be compared to chronic kidney disease, primarily resulting in decreased erythropoietin production, synthesized mainly in the kidneys (Grzelak and Fry 2022), contributing to anemia. Furthermore, intense parasitism may have also contributed to the hematological alterations observed, due to the inflammatory process triggered by the parasites in the urinary tract, confirmed by the presence of hematuria, leukocyturia, and bacteriuria, supporting the study by Pelligra et al. (2020), who reported hemorrhagic cystitis associated with secondary bacterial infection in a Persian cat and recurrent cystitis in a Pug dog caused by *Pearsonema sp.*

Renal function showed no alterations, as indicated by creatinine and urea levels, as well as by the urinary protein/creatinine ratio, which is consistent with the findings of Oliveira et al. (2021). In that study, the authors initially observed azotemia, which resolved following treatment, suggesting a pre-renal azotemia associated with dehydration. Despite *D. renale*’s ability to destroy the entire right renal parenchyma, leaving only the adult nematode retained in the fibrous capsule, it is possible for the maned wolf to survive with only one functional kidney (Leite et al. 2005; Hammond 2012).

The abdominal ultrasonographic examination revealed structures suggestive of *D. renale* adult worms within the right kidney and the right perirenal region, consistent with the observations of Oliveira et al. (2021). However, although less frequently, other organs may also be affected, including the left kidney (Perera et al. 2017). Despite the involvement of various organs, the right kidney is most frequently affected, possibly due to its proximity to the duodenum (Measures 2001).

Adult *Pearsonema sp.* worms are small, thread-like nematodes (males: 13–30 mm; females: 29–60 mm) that attach superficially or penetrate the bladder mucosa, making their detection by ultrasonography challenging (Petersen et al. 2018). This may explain why they were not visualized in the present study.

*Dioctophyma renale* has previously been reported in captive maned wolves as an important cause of mortality and has also been associated with poor reproductive performance (Dietz 1985). In this context, the laboratory findings observed in the present case are consistent with severe renal damage caused by dioctophymosis, reinforcing the potential pathological significance of this parasite in wild individuals.

Urinary capillariosis in this report is noteworthy, as infections are often asymptomatic or subclinical and are characterized by intermittent shedding of low numbers of eggs, which may hinder diagnosis; secondary cystitis, leukocyturia, and hematuria may also occur, as observed in the present case (Pelligra et al. 2020; Guimarães et al. 2020).

In the present study, egg morphology was consistent with that of *D. renale* and *Pearsonema* sp. The mean egg dimensions observed for *D. renale* (62–75 μm x 36–53 μm) were within the range reported by Pedrassani et al. (2017), and those of *Pearsonema sp.* (52–68 μm x 23–30 μm) were consistent with those described by Guimarães et al. (2020) and Pelligra et al. (2020).

To the best of our knowledge, this is the first report of a free-ranging maned wolf coinfection with *D. renale and Pearsonema* sp. in Latin America. Additionally, associated cystitis and complete destruction of the renal parenchyma were associated to parasitism. To date, there is only one previous record of capillariid parasitizing the urinary tract of a maned wolf, originating from Argentina (Beldomenico et al. 2002). Although *D. renale* has also been detected in this threatened species (Oliveira et al. 2021), coinfection with both parasites has not previously been documented. The findings reported in the present study were incidental; however, they reinforce that these parasitic infections may cause significant renal damage in this species and highlight the importance of including urinalysis in diagnostic protocols for wild animals. Importantly, this report describes laboratory findings and the diagnosis of dioctophymosis, findings consistent with severe renal damage. Further parasitological studies involving a larger number of free-ranging maned wolves are needed to assess the true prevalence and impact of these parasites on this threatened species in Brazil, as well as to improve strategies for early detection and clinical management.

## Data Availability

Not applicable.
